# Combined Inactivation of pRB and Hippo Pathways Induces Dedifferentiation in the *Drosophila* Retina

**DOI:** 10.1371/journal.pgen.1000918

**Published:** 2010-04-22

**Authors:** Brandon N. Nicolay, Battuya Bayarmagnai, Nam Sung Moon, Elizaveta V. Benevolenskaya, Maxim V. Frolov

**Affiliations:** 1 Department of Biochemistry and Molecular Genetics, University of Illinois at Chicago, Chicago, Illinois, United States of America; 2 Department of Biology, McGill University, Montréal, Québec, Canada; Harvard Medical School, Howard Hughes Medical Institute, United States of America

## Abstract

Functional inactivation of the Retinoblastoma (pRB) pathway is an early and obligatory event in tumorigenesis. The importance of pRB is usually explained by its ability to promote cell cycle exit. Here, we demonstrate that, independently of cell cycle exit control, in cooperation with the Hippo tumor suppressor pathway, pRB functions to maintain the terminally differentiated state. We show that mutations in the Hippo signaling pathway, *wts* or *hpo*, trigger widespread dedifferentiation of *rbf* mutant cells in the *Drosophila* eye. Initially, *rbf wts* or *rbf hpo* double mutant cells are morphologically indistinguishable from their wild-type counterparts as they properly differentiate into photoreceptors, form axonal projections, and express late neuronal markers. However, the double mutant cells cannot maintain their neuronal identity, dedifferentiate, and thus become uncommitted eye specific cells. Surprisingly, this dedifferentiation is fully independent of cell cycle exit defects and occurs even when inappropriate proliferation is fully blocked by a *de2f1* mutation. Thus, our results reveal the novel involvement of the pRB pathway during the maintenance of a differentiated state and suggest that terminally differentiated *Rb* mutant cells are intrinsically prone to dedifferentiation, can be converted to progenitor cells, and thus contribute to cancer advancement.

## Author Summary

The inability to respond to growth inhibitory cues is one acquired trait of a cancer cell. Almost all such signals are eventually routed through the Retinoblastoma (pRB) tumor suppressor pathway. Therefore, inactivation of the pRB pathway is considered to be an early and obligatory event during transformation of a normal cell into a malignant cancer cell. In this study, we found that inactivation of the Hippo pathway makes *Rb* mutant cells prone to undergo morphological changes and to become less differentiated, progenitor-like cells. Furthermore, we show that this was independent of the failure of *Rb* mutant cells to properly respond to cell cycle exit cues. These results are significant since, in general, tumors containing progenitor-like cells have a higher potential to progress through later stages of tumorigenesis and to become more aggressive and more deadly. Thus, the inactivation of *Rb* not only renders cells insensitive to growth inhibitory signals, but also sensitizes cells to revert to a progenitor-like state.

## Introduction

Almost all growth inhibitory signals ultimately act through the Retinoblastoma tumor suppressor protein (pRB) family [1]. In its active, hypophosphorylated form, pRB blocks cell proliferation by limiting the activity of the family of E2F transcription factors that control the expression of a large cohort of genes, including those that are essential for the G1 to S transition [2]. E2F activity is rate-limiting for S phase entry, as forced expression of E2F is sufficient to overcome the growth-inhibitory signals and drive quiescent or postmitotic cells into S phase [3]–[6]. Inactivation of pRB relieves the critical constraint from E2F, thus, rendering cells insensitive to antiproliferative signals, one of the acquired traits of a cancer cell. Indeed, the functional inactivation of the pRB pathway is believed to be an obligatory early step in the majority of human cancers [7]. Thus, the current paradigm posits that the tumor suppressive function of pRB is defined by its ability to promote cell cycle exit.

The view that pRB operates primarily during cell cycle exit is consistent with gene targeting studies in mice. Inactivation of the *Rb* gene family in mice resulted in ectopic proliferation and apoptosis [8], [9]; while the loss of *Rb* in quiescent or even in terminally differentiated cells led to inappropriate cell cycle re-entry [10], [11]. Interestingly, *Rb* knockout mice also exhibit reduced differentiation in multiple tissues suggesting that in addition to promoting cell cycle exit pRB may possess more specialized functions. In support of this idea, pRB was shown to directly interact with, and modulate activity of, cell-type specific transcription factors. For example, pRB interacts with the osteoblast transcription factor CBFA1/Runx2 and acts as a direct transcriptional co-activator of the CBFA1/Runx2 target genes required for the later stages of terminal differentiation [12]. This is particularly intriguing given the strong correlation between *Rb* mutations and the occurrence of osteosarcoma [13]. However, there is little support for the importance of these types of interactions *in vivo*. Further complicating the issue, several studies have demonstrated that some of the differentiation defects in *Rb* knockout mice are an indirect consequence of ectopic proliferation and apoptosis [14]–[16]. These results highlight the necessity to determine the *bona fide* role of *Rb* in promoting differentiation *in vivo*.

*Drosophila* represents a powerful alternative model system to study the *in vivo* function of the pRB pathway, since the homologous E2F and pRB (termed RBF) gene families are smaller in flies than in mammals, yet their involvement in cell cycle control is remarkably conserved [17]. Curiously, the consequences of the loss of *rbf* in somatic tissues such as the larval eye imaginal disc are rather subtle [18]–[20]. One possibility is that other pathways may mask otherwise critical functions of *rbf* and thus compensate for its loss. This is an important conceptual point, since in addition to the inactivation of the pRB pathway, a cancer cell acquires mutations in multiple tumor suppressors and oncogenes; and the collective outcome of these alterations eventually determines the malignancy of the cancer cell [7].

The recently identified Hippo tumor suppressor pathway represents an attractive candidate for such a role in compensation, since like the pRB pathway it regulates cell cycle exit (for review see: [21]–[23]). In the center of the Hippo pathway is a kinase cascade that contains Hippo (Hpo) and Warts (Wts). Wts is the most downstream kinase of the cascade; and additionally the Fat pathway controls the level and activity of Wts. A well-known function of Wts in both pathways is to negatively regulate the transcriptional co-activator, Yorkie (Yki). Inactivation of the Hippo kinase cascade by mutations of *hpo* or *wts*, or overexpression of *yki*, increases the rate of cell duplication during the growth phase of imaginal discs, protects cells from apoptosis, and delays the cell cycle exit of the uncommitted interommatidial cells of the larval eye imaginal disc. Although the Hippo pathway is best known for its role in regulation of cell proliferation, there is increasing evidence of its functions in postmitotic cells [24]–[26].

Classically, the *Drosophila* eye imaginal disc has been used to study the results following the inactivation of the Hippo and pRB pathways *in vivo*. A key reason is that in the eye disc cell proliferation and differentiation occur in highly reproducible patterns which are established through the developmentally regulated movement of the morphogenetic furrow (MF) [27]. In this model system even small perturbations in terminal cell cycle exit or differentiation can dramatically alter eye development and can therefore be unambiguously characterized.

**Figure 1:**
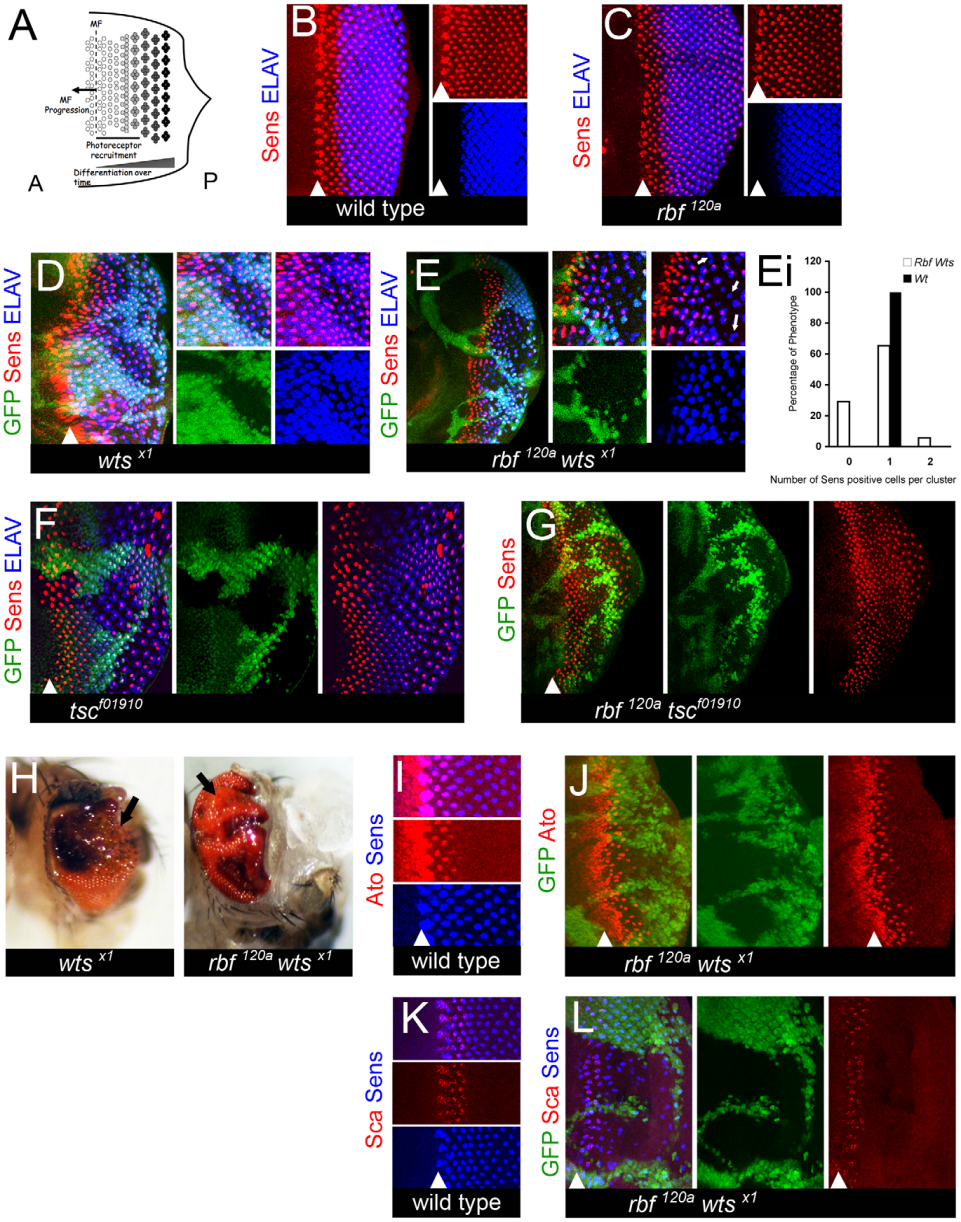
*rbf wts* double mutants have defects in differentiation. (A) Schematic of the spatiotemporal model of differentiation during larval eye development. The morphogenetic furrow [(MF), dotted line] moves from the posterior (P) edge of the disc towards the anterior (A) compartment. As it moves into contact with cells they begin to undergo a progressive differentiation that occurs in stages. The first being a progressive recruitment period to develop cell identity (marked by bar); the second stage being a cell autonomous program to complete terminal neuronal differentiation and maintain mature neuronal identity. Therefore, the cells in the most posterior regions of the disc have been fully differentiated the longest time and those cells nearest to the MF are at the earliest stages of differentiation. (B–L) The position of the MF is shown by a white arrowhead and the posterior compartment is to the right in all images. All images of larval discs are projection images. Clones of mutant cells were induced with the FLP-FRT system and distinguished by the lack of GFP (green). (B–G) Eye discs were labeled for the Senseless (Sens) protein in red and the Elav protein in blue. (I,J) Eye discs were labeled for the Senseless (Sens) protein in blue and the Atonal (Ato) protein in red. (K,L) Eye discs were labeled for the Senseless (Sens) protein in blue and the Scabrous (Sca) protein in red. (B) A wild-type disc. (C,D) Photoreceptors differentiate normally in the eye disc hemizygous for the *rbf^120a^* mutant allele (C) and in clones of *wts^x1^* mutant cells (D). (E) The number of Sens positive cells is reduced in the posterior of the *rbf^120a^ wts^x1^* mutant clones. White arrows point to Elav positive clusters of cells that lack any Sens positive cell. Elav expression reveals an incomplete complement of photoreceptors in the posterior of the double mutant tissue. (Ei) Quantification of Sens positive cells present in *rbf^120a^ wts^x1^* mutant clones (see Materials and Methods for details). A Student's *t*-test revealed that the differences between the *rbf^120a^ wts^x1^* double mutant cells and the wild-type population of Elav positive cells with zero or two Senseless positive cells were statistically significant with a *p*-value <0.05. (F,G) The loss of differentiation is specific to a genetic interaction between the pRB and Hippo pathways as photoreceptors differentiate normally in (F) *tsc^f01910^* and (G) *rbf^120a^ tsc^f01910^* mutant clones. Loss of *tsc* results in larger than normal cells, thus leading to increased spacing between adjacent ommatidia marked by Sens and Elav. (H) *wts^x1^* single mutant and *rbf^120a^ wts^x1^* double clones were generated at a low frequency with *hs*-FLP and examined in the adult eye. Arrows highlight the differences between the two tissue samples shown. The *wts^x1^* single mutant tissue is well differentiated and contains a high number of bristles (black dots). In contrast, the surface of the *rbf^120a^ wts^x1^* double mutant tissue is glossy and lacks bristles. (I) A wild-type eye disc. Senseless expression in pre-R8 cells requires that Atonal expression be initiated first. After the expression of Senseless has been able to define the R8 cell and to begin recruitment of the ensuing R cells, Atonal expression is lost. (J) Atonal expression is normal in the *rbf^120a^ wts^x1^* double mutant tissue suggesting that mature R8 cells expressing Senseless can develop normally. (K) A wild-type eye disc. Ommatidial cluster formation relies upon proper spacing to be present between clusters. This spacing is defined early in the recruitment and refinement stage of differentiation of the R8 cell when a Senseless positive cell expresses the glycoprotein Scabrous. (L) Scabrous expression appears normal in *rbf^120a^ wts^x1^* double mutant tissue. Therefore, *rbf^120a^ wts^x1^* mutant R8 cells can successfully be refined and can establish proper spacing for further recruitment of the ensuing R cells to take place.

The adult eye is composed of a regular array of identical units termed ommatidia. Each individual ommatidium contains a cluster of eight photoreceptors, termed R1 through R8. The passage of the MF results in specification of the pre-R8 photoreceptors that act as the founder cell of each ommatidium (Figure 1A). Once an R8 is selected, the recruitment and selection of all other photoreceptors occurs in a strict procession through reiterative use of the epidermal growth factor receptor (EGFR) pathway [28]. As each R cell is specified it undergoes terminal differentiation, develops an axonal projection, and through expression of cell type specific factors eventually becomes a fully mature photoreceptor. Since the selection, specification, and ensuing maturation of all R cells occurs continuously with respect to the position of the MF, then the photoreceptors that are in the earliest stages of differentiation are always found within and immediately posterior to the MF (Figure 1A). It then follows that the very first photoreceptors to completely differentiate can be found in the most posterior regions of the disc (Figure 1A). This regimented developmental program provides a unique spatiotemporal model to visualize all steps of photoreceptor recruitment and subsequent differentiation in the same eye disc.

We therefore utilized the *Drosophila* eye imaginal disc to examine the impact of the combined inactivation of the pRB and Hippo pathways. We found that *rbf wts* or *rbf hpo* double mutant cells initiate and progress through the neuronal differentiation program. However, double mutant cells failed to maintain their neuronal identity, dedifferentiated, and became uncommitted eye specific cells. Dedifferentiation of *rbf wts* double mutant photoreceptors was accompanied by widespread inappropriate proliferation. Yet, the two defects were independent of each other as *rbf wts* mutant photoreceptors dedifferentiated even when inappropriate proliferation was fully blocked by a *de2f1* mutation. Thus, our findings suggest that the pRB pathway in cooperation with the Hippo pathway plays a specific role in maintenance of the differentiated state that is distinct from the pRB function to promote cell cycle exit.

## Results

### Inactivation of the Hippo pathway results in progressive loss of neuronal markers in differentiating *rbf* mutant cells

As described above, the *Drosophila* eye provides a unique experimental system in which differentiation and proliferation during development can be readily studied (Figure 1A). We took advantage of this well-characterized spatiotemporal model and carefully examined the properties of *rbf* mutant cells following inactivation of the Hippo pathway.

We began our analysis by examining neuronal differentiation of R8 photoreceptors. Because R8 is the founder cell for each ommatidium, its recruitment is independent of the correct specification of the remaining photoreceptors in the ommatidium. In contrast, other photoreceptor recruitment occurs progressively in a stepwise manner (R2/R5 followed by R3/R4, R1/R6 and finally R7) and is dependent upon the presence of the previously recruited pair of R cells [28].

R8 photoreceptors can be uniquely identified by the expression of the transcription factor Senseless (Sens) [29] (Figure 1B) that is first detected immediately posterior to the MF. As photoreceptors progressively differentiate, they begin to express a late neuronal marker, Elav, several columns posterior to the onset of Sens expression (Figure 1B). Unlike Sens, Elav is expressed in all R cells. Therefore in a wild-type disc, each cluster of Elav positive cells contains a single Sens positive cell (Figure 1B).

As previously reported [19], the pattern of Sens and Elav expression was relatively normal in an *rbf* mutant disc, although there were slight abnormalities of Sens expression within cells immediately adjacent to the MF (Figure 1C). To examine the expression of these differentiation markers in Hippo pathway mutant cells, we employed the FLP-FRT technique to generate clones of homozygous *wts* mutant cells. In this technique, homozygous wild-type cells are marked with GFP, while homozygous *wts* mutant cells are distinguished by the lack of GFP. In spite of increased spacing between adjacent ommatidia in *wts* mutant clones, each ommatidium (marked by Elav) still contained a single R8 cell (marked by Sens) (Figure 1D). The increased spacing between ommatidia is due to inappropriate proliferation of non-neuronal, interommatidial cells that have failed to exit the cell cycle. Thus, consistent with previous reports (for example see: [18], [19], [30]–[32]), neither the loss of *rbf* nor the loss of Hippo pathway signaling affected photoreceptor differentiation.

To determine the effect of inactivation of Hippo pathway signaling in *rbf* mutant cells, we examined clones of *wts* mutant cells generated in hemizygous *rbf^120a^* mutant eye discs. Expression of Sens was properly initiated as *rbf wts* double mutant cells emerged from the MF. However, the number of Sens positive cells was severely decreased toward the posterior of the mutant clones (Figure 1E). Additionally, we noted that approximately one third of the ommatidial clusters, as visualized by Elav expression, were missing Sens positive R8 cells (examples are pointed to by arrows in Figure 1E and quantification is presented in Figure 1Ei). To exclude the possibility of allele specific effects, we confirmed our findings with a *hpo^MGH4^* mutant allele (Figure S1) and with an *rbf^14^* allele (Figure S2). We note, that the phenotype of the *rbf^14^ wts^x1^* double mutant cells was more severe than that in the *rbf^120a^ wts^x1^* double mutant tissue. Since *rbf^14^* is a null allele, while a small amount of the RBF protein is produced from the *rbf^120a^* allele [19], this suggests that the double mutant phenotype is highly sensitive to the dosage of RBF. Thus, we concluded that following specification mature R8 cells were gradually lost as neuronal differentiation proceeded. Importantly, the progressive loss of Sens expression in *rbf* mutant cells is specific to inactivation of Hippo signaling since a mutation in another tumor suppressor, *tsc1* [33], has no effect on R8 differentiation in *rbf tsc1* double mutant cells (Figure 1F and 1G and data not shown). Consistently with the larval eye disc analysis, in the adult eye, the *rbf wts* mutant clones had a characteristic glossy appearance that is indicative of a lack of differentiated photoreceptors (Figure 1H).

The loss of Sens expression in *rbf wts* double mutant tissue could be due to a failure to properly specify R8 cells. Therefore we examined expression of the proneural gene *atonal* (*ato*) since its expression pattern defines the pre-R8 cell recruitment and early specification of a mature R8 cell. In a wild-type disc, Ato is initially expressed in all cells of the MF. Later, Ato expression is resolved to a single pre-R8 cell via proneural enhancement and lateral inhibition [34] (Figure 1I). In clones of *rbf wts* double mutant cells, Ato expression is initiated and refined to a single cell properly indicating that specification of R8 cells occurs normally (Figure 1J). To further support this conclusion, we examined expression of the fibrinogen-related protein Scabrous (Sca), another useful marker of R8 cell development. Sca is required for the restriction of Ato expression during the process of lateral inhibition that occurs in the resolution of a mature R8 cell [35]. As shown in Figure 1K and 1L, the pattern of Sca expression remains largely unaffected in the *rbf wts* double mutant tissue. We concluded that following developmental specification and refinement, mature R8 cells are progressively lost in *rbf wts* double mutant tissue. This is in striking contrast to the phenotypes of *rbf, hpo*, or *wts* single mutants, in which following specification and refinement, differentiation of photoreceptors remains normal.

### The progressive loss of differentiation markers is not due to the elimination of cells by apoptosis

**Figure 2:**
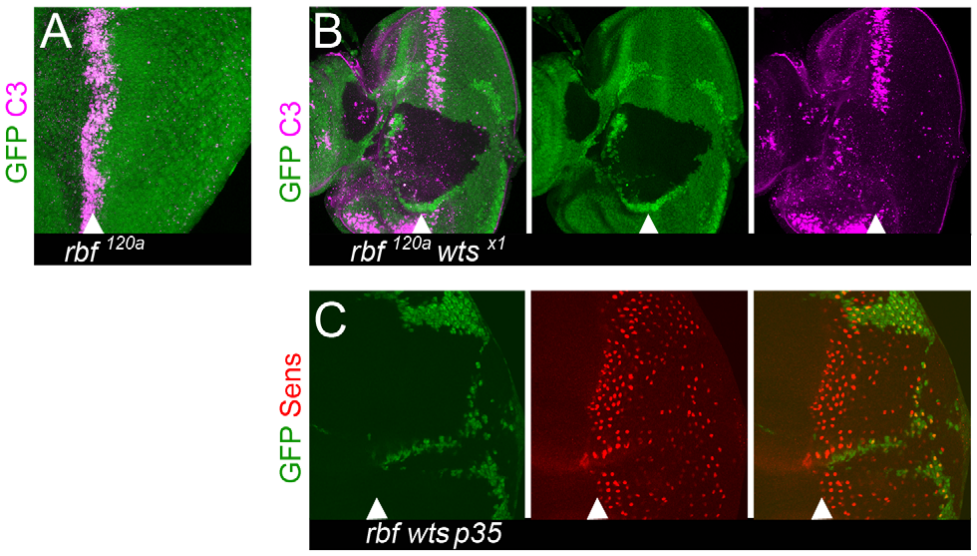
Loss of differentiation markers is not due to apoptosis. Apoptosis does not contribute to the reduction in the number of Sens positive cells. Clones of mutant cells were induced with the FLP-FRT technique and distinguished by the lack of GFP (green). (A) Apoptosis, as detected by the cleaved caspase-3 (C3) antibody (magenta), in the MF of a disc hemizygous for *rbf^120a^* but not in the posterior compartment. (B) Lack of cleaved caspase-3 positive cells, as detected by the C3 antibody (magenta), in clones of *rbf^120a^ wts^x1^* double mutant cells within and posterior to the MF. (C) Overexpression of p35 within and posterior to the MF does not rescue the reduced number of Sens positive cells in the posterior of *rbf^120a^ wts^x1^* mutant clones.

A simple explanation for the progressive reduction in the number of Sens positive cells within the *rbf wts* double mutant tissue is that these cells are eliminated through apoptosis. To test this idea, we used an antibody that recognizes a cleaved form of caspase 3 (C3) to visualize apoptotic cells. The loss of *rbf* leads to a significant level of apoptosis immediately posterior to the MF (Figure 2A) [19]. Strikingly, a *wts* mutation protects *rbf* deficient cells from apoptosis as no C3 positive cells were found within *rbf wts* double mutant tissue including the region posterior to the MF, where differentiation occurs (Figure 2B). The lack of apoptotic cells in *rbf wts* double mutant tissue is consistent with the known ability of mutations in the Hippo pathway to protect cells from several types of apoptosis [31], [32], [36].

To further confirm that apoptosis does not contribute to the loss of Sens expressing *rbf wts* mutant cells we overexpressed the baculovirus protein p35 in all cells posterior to the MF. p35 is a caspase inhibitor that potently blocks most cell death in *Drosophila* [37]. As shown in Figure 2C, the number of R8 cells, visualized by the expression of Sens, were progressively lost in the posterior region of the *rbf wts* double mutant clone even when p35 was overexpressed. We therefore concluded that a progressive reduction in the number of Sens positive cells is because *rbf wts* double mutant cells failed to maintain the neuronal differentiated state.

### *rbf wts* double mutant photoreceptors stochastically dedifferentiate

**Figure 3:**
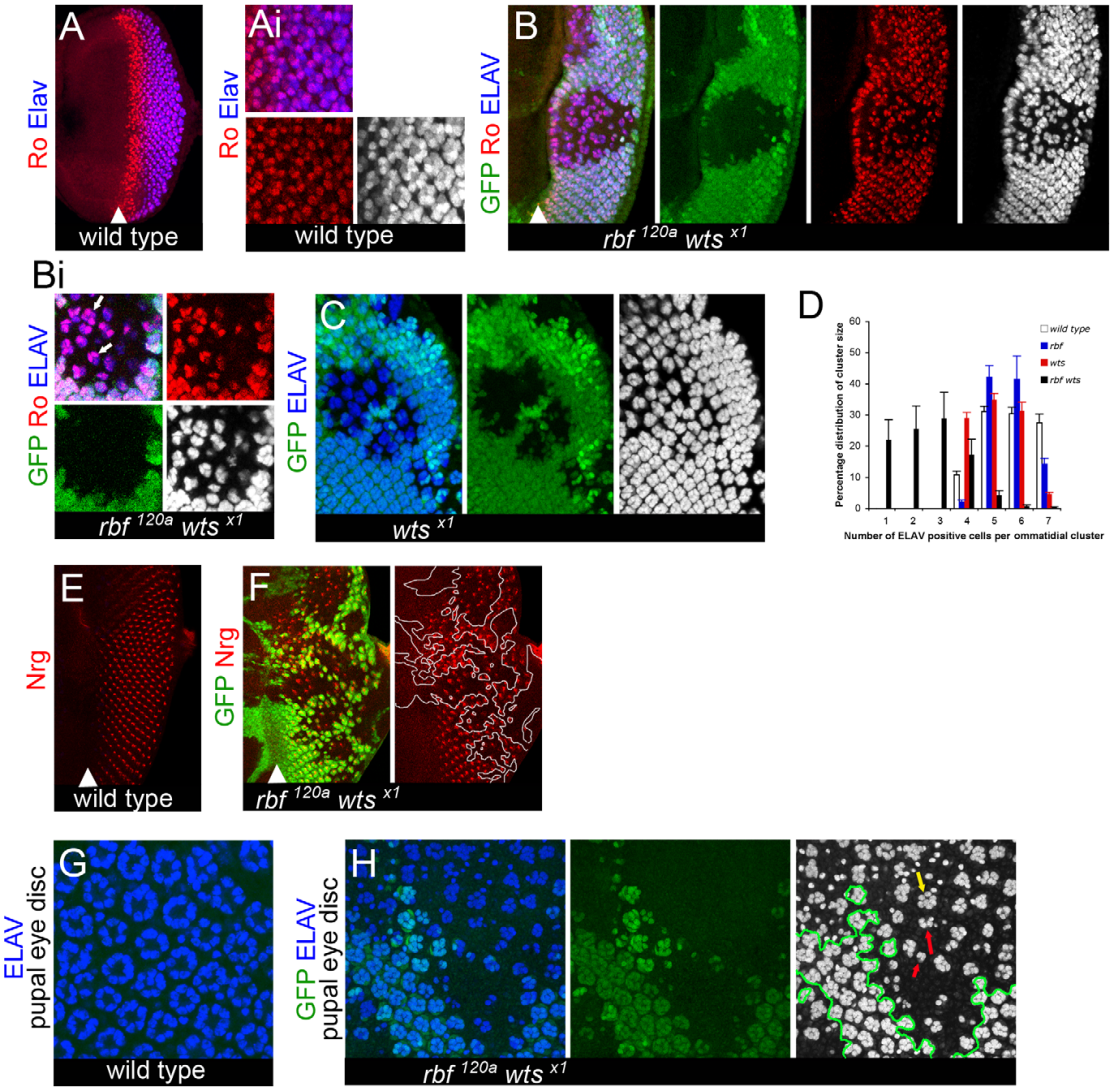
Dedifferentiation of *rbf wts* double mutant cells. Clones of mutant cells were induced with the FLP-FRT technique and distinguished by the lack of GFP (green). In all merged images Elav is blue, and when shown on a single channel it is on the gray scale. All images are projection images. (A–Ai) Expression of the protein Rough (Ro) (red) is detected at high levels in R2/5 and at lower levels in R3/4 cells in a wild-type disc. Elav expression is found in all mature R cells. Rough positive cells are developed and recruited by the proper refinement and resolution of R8 cells. Once developed, Rough positive cells take on a cell autonomous program to maintain cell identity. (B–Bi) The correct numbers (shown by white arrows in Bi) of Ro (red) positive cells are resolved in *rbf^120a^ wts^x1^* double mutant ommatidial clusters marked by Elav nearest the MF. However, a stochastic pattern of ommatidial cluster appearance begins further posterior from the MF, seen by the loss of both Ro and Elav expression in the *rbf^120a^ wts^x1^* double mutant clones. (C) This is in contrast to the ommatidial cluster development and the ability of the ommatidial cells to maintain proper identity (shown by Elav expression in *wts^x1^* single mutant clones). (D) Quantification of the number of Elav positive cells within a mature photoreceptor cluster in four genotypes (wild-type, *rbf1^120a^, wts^x1^* and *rbf1^120a^ wts^x1^*) (see Materials and Methods for details). Counting was done from the third Elav positive column behind the MF to the posterior edge, an area in a wild-type eye disc that will have developed a range of either 5–7 detectable Elav positive cells per cluster. Error bars are standard deviations from the mean of each category per genotype. A Student's *t*-test between each mutant genotype and the wild-type population revealed that no statistical difference between the *rbf* mutant and wild-type tissue existed. A *p*-value <0.05 existed between the *wts* mutant and wild-type tissue for the number of ommatidial clusters with 7 Elav positive cells. A *p*-value <0.05 existed between the *rbf wts* double mutant an wild-type for the number of ommatidial clusters with 1, 2, or 3 Elav positive cells and a *p*-value <0.01 for the number of ommatidial clusters with 5, 6, or 7 Elav positive cells. We note that the distribution of the defects in differentiation are not directly due to developmental recruitment as we can find complete and incomplete ommatidial clusters in the *rbf wts* double mutant eye disc (see distribution of percentages). (E,F) Expression of the late neuronal marker Neuroglian (Nrg) (red) in a wild-type disc (E). *rbf^120a^ wts^x1^* double mutant cells initially express the late neuronal marker Neuroglian (Nrg) (red), but then lose expression of Nrg in the posterior regions of the disc (F). (G,H) Wild-type expression of Elav in pupal retinas (44–48hr APF) reveals that Elav expression is not recovered later in development of *rbf^120a^ wts^x1^* double mutant photoreceptor cells (mutant tissue is separated from wild-type tissue by green outline on gray scale). A variable number of Elav positive cells, from normal (yellow arrow) to highly reduced (red arrows), can be found amongst each *rbf wts* double mutant ommatidium in (H).

Because defects in R8 differentiation are known to prevent development of other photoreceptors, we examined the differentiation of other R cells in *rbf wts* double mutant tissue. R2/R5 are recruited following R8 specification and can be identified by the high level of expression of the transcription factor Rough (Ro); that is also expressed at a lower level in R3/R4, which develop following R2/R5 (Figure 3A and 3Ai). Despite the stochastic disappearance of R8 cells in *rbf wts* double mutant tissue, one could find mutant ommatidia containing the correct number of two cells per cluster highly expressing Ro, thus indicating that recruitment of R2/R5 cells can occur properly (pointed to by arrows in Figure 3Bi). However, there were ommatidia that had either only a single or no Ro expressing cells. Furthermore, the number of Ro positive cells was generally reduced in the posterior region of the clone (Figure 3B and 3Bi and Figure S3). These results suggested that R2/R5 development could occur normally, but that like the defects seen in R8 cells, following specification could not always be maintained as mature photoreceptors.

Consistent with the notion that mature R cells were being lost we found that the total number of photoreceptors per ommatidium was highly variable in the *rbf wts* double mutant tissue as revealed by expression of Elav, which visualizes all photoreceptors in the ommatidium (Figure 3B and 3C). Quantification supported the conclusion that there was not a stage in recruitment and specification of R cells that appeared to be completely inhibited (Figure 3D) suggesting that the defects seen were not directly due to developmental signaling being affected.

In spite of the stochastic loss of neuronal markers, the cellular program of terminal neuronal differentiation was not blocked, as *rbf wts* double mutant photoreceptors retained the ability to complete neuronal differentiation. In wild-type discs, as photoreceptors differentiate they begin to express the neuron specific form of neuroglian (Nrg) along axonal projections which can be detected with the BP104 antibody (Figure 3E) [38]. These axonal projections migrate to connect with the optic lobe to form functional light sensory cells. We observed that *rbf wts* double mutant cells express Nrg in the anterior region of mutant clones (Figure 3F); indicating that as *rbf wts* mutant photoreceptors differentiate they exhibit characteristic morphological features of normal photoreceptors at this stage of development. However, similar to the expression of Elav, Ro, and Sens, expression of Nrg largely disappeared in the posterior region of the mutant clone.

To determine if the defects in larval eye differentiation could be corrected later in development, we examined mosaic pupal eye discs at a stage when all cells of a mature ommatidium (including pigment, cone, and bristle cells) have developed. We found that there were fewer ommatidia, as revealed by Elav expression, in the *rbf wts* double mutant tissue than in the adjacent wild-type tissue and that mutant ommatidia contained a reduced number of photoreceptors (Figure 3G and 3H). We concluded that the failure of *rbf wts* double mutant cells to maintain a differentiated state was not limited to R8 cells, appeared to occur stochastically, and therefore reflects a general requirement of the pRB and Hippo pathways in maintenance of neuronal differentiation.

**Figure 4:**
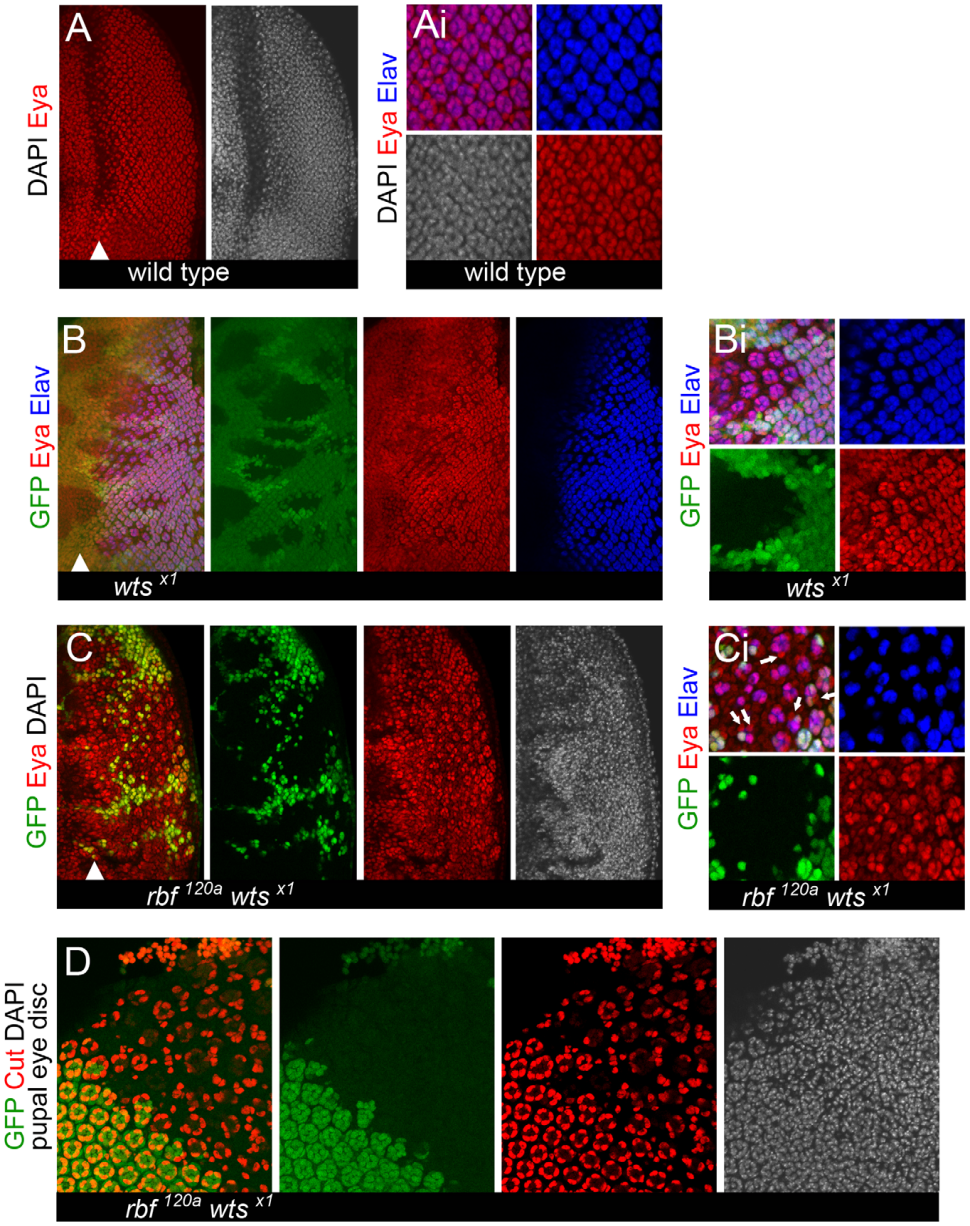
Expression of the eye specification factor Eyes Absent (Eya) in dedifferentiating *rbf wts* double mutant photoreceptors. Clones of mutant cells were induced with the FLP-FRT system and distinguished by the lack of GFP (green). (A–Ai) Expression of the eye specification factor Eyes Absent (Eya) (red) and the neuronal specific protein Elav (blue) in a wild-type disc. Eya expression is highest in mature, differentiated cells, marked by Elav (blue). (B–Bi) Loss of *wts* does not affect the expression pattern of Eya. The reduced expression of Eya (red) in population of the unspecified interommatidial cells between ommatidial clusters (marked by Elav (blue)) can be seen more easily in a *wts* single mutant background (Bi). (C) Eya is expressed in *rbf^120a^ wts^x1^* double mutant cells. (Ci) Examples of *rbf^120a^ wts^x1^* double mutant cells adjacent to ommatidial clusters can frequently be found where cells no longer express Elav but have a high level of Eya (pointed by arrows). (D) *rbf^120a^ wts^x1^* double mutant cells do not transdifferentiate into cone cells (Cut expression is in red) later in development (44–48 hr APF).

To further characterize the progressive loss of differentiated cells in the *rbf wts* double mutant tissue we examined the expression of the eye determination gene *eyes absent* (*eya*). Eya promotes differentiation and plays a major role in the transcription factor network that controls eye development [39]. In wild-type eye discs, Eya is expressed at a high level in differentiating photoreceptors while uncommitted interommatidial cells express Eya at a lower level (Figure 4A and 4Ai) [39]. This difference in the levels of Eya is especially evident in clones of *wts* mutant cells in which the population of interommatidial cells is expanded (Figure 4B and 4Bi). Eya remains to be expressed throughout the *rbf wts* double mutant tissue even in the most posterior regions, where loss of differentiation markers was most often observed (Figure 4C and 4Ci). As shown in Figure 4Ci, Eya was highly expressed in *rbf wts* double mutant photoreceptors while interommatidial cells had a low level of Eya. Interestingly, we observed numerous examples of Elav negative *rbf wts* double mutant cells with a high level of Eya (pointed to by arrows in Figure 4Ci). Since these cells were found immediately adjacent to ommatidial clusters it is attractive to speculate that these cells are undergoing dedifferentiation. However, this could not be directly tested due to the lack of markers of dedifferentiated cells. Nevertheless, given that the eye determination gene Eya was expressed in all cells within the *rbf wts* double mutant tissue we concluded that cells no longer expressing differentiation specific proteins did not change their identity and remained eye specific cells.

To determine whether dedifferentiated *rbf wts* double mutant photoreceptors eventually differentiate into different cell types such as cone cells we examined expression of the cone cell marker Cut in mosaic pupal eye discs. At 48 hr after puparium formation, four cone cells per ommatidium were present in wild-type tissue (Figure 4D). In contrast, the number of cone cells per ommatidium was reduced in *rbf wts* double mutant tissue. In general, we observed one to three cone cells, although one could also find ommatidia containing precisely four cone cells per ommatidium (Figure 4D). However, we did not find any indication that cone cells overpopulate the mutant tissue suggesting that *rbf wts* double mutant cells do not transdifferentiate into cone cells.

Taken together these data suggest that once recruited and specified *rbf wts* double mutant photoreceptors properly initiate and progress through the neuronal differentiation program. However, over time *rbf wts* double mutant photoreceptors stochastically lose their morphological features and become undifferentiated, eye specific cells. Therefore, we concluded that *rbf wts* double mutant photoreceptors undergo dedifferentiation.

### *rbf wts* double mutant cells bypass terminal cell cycle exit signals

**Figure 5:**
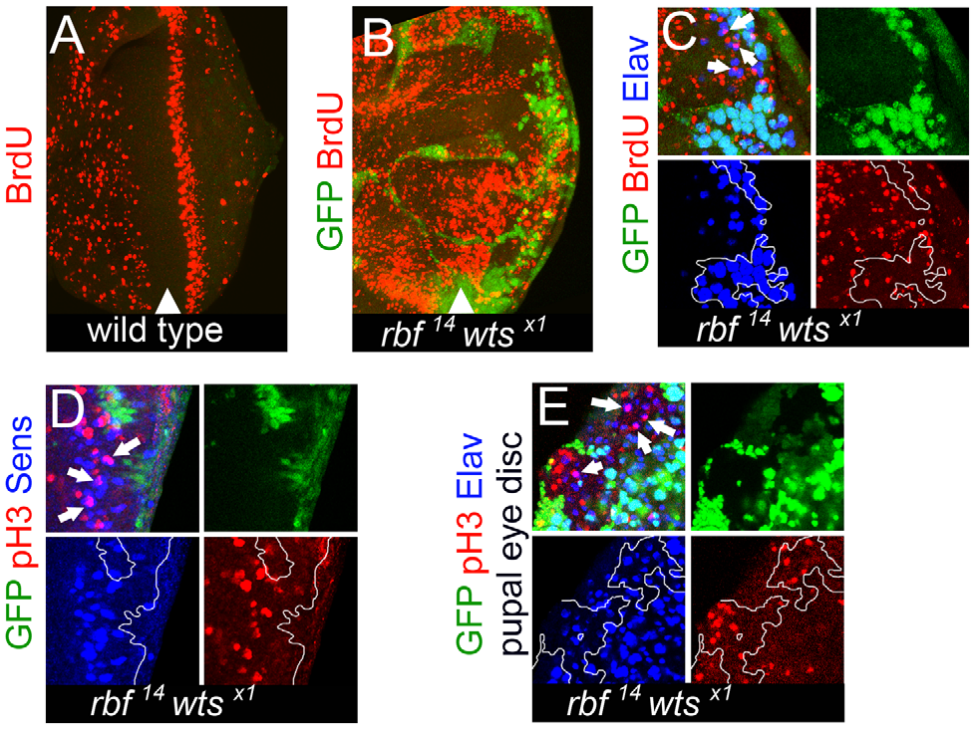
*rbf wts* double mutant cells fail to exit the cell cycle while undergoing photoreceptor differentiation. S phase cells in the eye disc were revealed by BrdU labeling. (A) No BrdU positive cells were found posterior to the second mitotic wave (SMW) in a wild-type disc. (B) Inappropriate S phases posterior to the SMW in the *rbf^14^ wts^x1^* double mutant tissue. (C) Differentiated *rbf^14^ wts^x1^* double mutant cells (Elav) (blue) were undergoing S phases (pointed by arrows). (D) *rbf^14^ wts^x1^* double mutant cells expressed the R8 marker Sens (blue) even during mitosis (marked by pH3) (red). Examples are pointed by arrows. (E) *rbf^14^ wts^x1^* double mutant photoreceptors (Elav) (blue) continued to undergo mitosis (marked by pH3) (red) in pupal eye discs.

In normal cells proliferation and differentiation are tightly coordinated and are generally incompatible with each other. Dedifferentiation in *rbf wts* double mutant tissue prompted us to examine the impact of the combined loss of *rbf* and *wts* during cell cycle exit. We used BrdU labeling to mark cells in S phase. In the larval eye disc, the pattern of cell proliferation is linked to the MF, where cells are arrested in G1 and therefore do not incorporate BrdU (Figure 5A). Directly posterior to the MF, uncommitted cells undergo a synchronous round of the cell cycle called the second mitotic wave (SMW). No proliferation occurs posterior to the SMW as all cells withdraw from the cell cycle. *rbf* mutant eye discs exhibit only minor perturbations in cell cycle exit (for example: [19] and data not shown). Inactivation of the Hippo pathway leads to inappropriate proliferation of interommatidial cells posterior to the SMW while photoreceptors exit the cell cycle properly ([31], [32], [36] and data not shown). Not surprisingly, the precise pattern of cell proliferation was completely lost in *rbf wts* double mutant tissue. *rbf^14^ wts^x1^* double mutant cells were found to be inappropriately undergoing S phases and subsequent mitoses within the MF and posterior to the SMW (Figure 5B–5D). Unexpectedly, we found that in addition to interommatidial cells, these inappropriate cell divisions were taking place in fully differentiated cells, marked by Elav and Sens expression (Figure 5C and 5D), a phenotype that is distinct from either the loss of *rbf* or *wts* alone [19], [31], [32], [36]. At least some of these differentiated cells continued to proliferate during early pupal development as revealed by the occurrence of lone Elav positive cells which expressed the mitotic marker phosphorylated histone H3 (pH 3) in confocal images (Figure 5E). In summary, these results show widespread inappropriate proliferation of differentiated *rbf wts* double mutant photoreceptors.

### Inappropriate proliferation does not induce dedifferentiation of *rbf wts* double mutant cells

**Figure 6:**
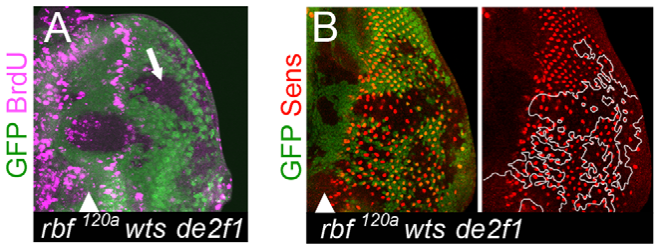
Cells lacking *rbf* and *wts* fail to maintain the differentiated state even in the absence of inappropriate proliferation. (A) *rbf^120a^ wts^x1^ de2f1^729^* triple mutant cells failed to proliferate posterior to the SMW as revealed by BrdU labeling. (B) The number of Sens positive cells remained reduced in the posterior of the *rbf^120a^ wts^x1^ de2f1^729^* triple mutant tissue.

In several experimental systems, when a differentiated cell reenters the cell cycle it undergoes dedifferentiation. Thus, dedifferentiation in *rbf wts* double mutant clones could be induced by inappropriate reentry into the cell cycle. To test this idea, we examined the procession of differentiation in *rbf wts* double mutant tissue when inappropriate proliferation of these cells was blocked by a *de2f1* mutation. In agreement with our previous findings that *de2f1* is specifically required during inappropriate proliferation *wts* mutant cells [40], *rbf wts de2f1* triple mutant cells failed to incorporate BrdU posterior to the SMW, but not in the anterior compartment when cells are normally cycling asynchronously (Figure 6A). However, even in the complete absence of inappropriate proliferation we observed a widespread dedifferentiation in the *rbf wts de2f1* triple mutant tissue as evidenced by the progressive loss of Sens positive cells in the posterior region (Figure 6B). As in the *rbf wts* double mutant clones, cell death that might account for the loss of Sens positive cells was not detected in *rbf wts de2f1* triple mutant tissue posterior to the MF (Figure S4). Additionally, *wts* mutant cells are intrinsically protected from apoptosis due to Yki induced upregulation of the *Drosophila inhibitor of apoptosis*, *diap1*; indeed, *diap1* remained induced in the triple mutant cells (Figure S4). Thus, inappropriate proliferation of *rbf wts* double mutant photoreceptors does not induce their dedifferentiation. Furthermore, since the loss of *de2f1* had no effect on the *rbf wts* mutant phenotype this suggests that dedifferentiation occurs in a dE2F1 independent manner and, thus, reflects a dE2F1 independent function of RBF.

### *yki* is insufficient to induce dedifferentiation in *rbf* mutant photoreceptors

**Figure 7:**
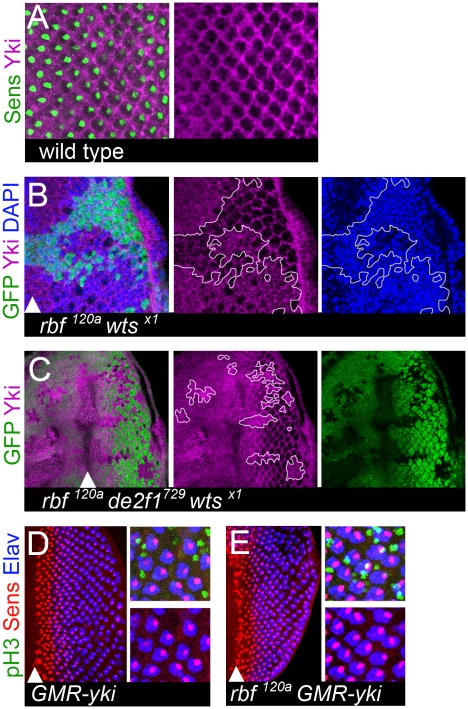
Aberrant Yki activity is not sufficient to trigger dedifferentiation of *rbf* mutant cells. (A) In the posterior of the wild-type eye imaginal disc, Yki (magenta) is mainly present in interommatidial cells and excluded from differentiating photoreceptors (marked by Senseless (green)). (B,C) Yki is localized in the nuclei (marked by DAPI (blue)) of *rbf^120a^ wts^X1^* double mutant cells (B) and *rbf^120a^ e2f1^729^ wts^X1^* triple mutant cells (C). Clones of mutant cells are marked by the lack of GFP and outlined. (D,E) Expression of a constitutively active Yki^S168A^ in the posterior compartment of a wild-type eye disc using a *GMR*-Gal4 driver drives inappropriate cell divisions of interommatidial cells (pH3 (green)), but has no effect upon differentiated ommatidial cells (Elav (blue), Senseless (red)) (D). In contrast, expression of a constitutively active Yki^S168A^ in the posterior compartment of a *rbf^120a^* hemizygous disc using a *GMR*-Gal4 does drive postmitotic photoreceptors into the cell cycle, as seen by co-localization of pH3 with Sens and Elav positive cells (white). However, this does not cause dedifferentiation of *rbf* mutant cells since cells still retain expression of both Sens and Elav in the most posterior regions (E).

Yki represents a critical effector of the Hippo pathway and mediates its growth output. In *wts* or *hpo* mutants, Yki inappropriately translocates to the nucleus and activates Hippo pathway target genes that promote cell proliferation and block apoptosis [41]. Therefore we examined the subcellular localization of Yki in *rbf wts* double mutant cells. Yki is mostly cytoplasmic in wild-type cells, while it becomes more nuclear in *wts* mutant cells [42], [43]. In the posterior region of developing larval eye imaginal disc, Yki was largely present in interommatidial cells and was undetected in differentiated photoreceptors (Figure 7A). In contrast, Yki was localized to both the cytoplasm and nucleus in *rbf wts* double mutant cells (Figure 7B). This was not a result of inappropriate proliferation, since Yki remained primarily nuclear in *rbf wts de2f1* triple mutant cells (Figure 7C) that did not proliferate posterior to the SMW (Figure 6A). Thus, we concluded that Yki is inappropriately localized to the nucleus in both *rbf wts* double mutant cells and in *rbf wts de2f1* triple mutant cells, which along with dIAP1 upregulation (Figure S4), is a hallmark of Yki activation.

Next, we tested whether activation of Yki is sufficient to trigger dedifferentiation of *rbf* mutant photoreceptors. We overexpressed a hyperactive form of Yki^S168A^ using a *GMR-Gal4* driver in cells posterior to the MF in the *rbf^120a^* mutant eye disc and analyzed the expression of Sens and Elav. The *GMR-Gal4* driver has been previously validated in experiments to assess the function of Yki and its mammalian homolog YAP (for example see: [44]). Yki^S168A^ is resistant to an inhibitory phosphorylation by the Wts kinase and therefore more efficiently translocates to the nucleus [42], [43]. Overexpression of Yki resulted in excessive proliferation in the posterior part of both the wild-type and *rbf* mutant eye disc (Figure 7D and 7E). This led to an increased spacing between adjacent ommatidial clusters. Surprisingly, in contrast to *rbf wts* or *rbf hpo* double mutant cells, overexpression of Yki did not induce dedifferentiation of *rbf* mutant cells, as there was no reduction in the number of Elav and Sens positive cells even in the most posterior region (Figure 7E). We note however that there is a spatial difference in Yki activation in these two settings. In *rbf wts* and in *rbf hpo* double mutant clones activation of Yki occurred prior to the MF while the *GMR-Gal4* driver induced Yki expression in cells within and posterior to the MF. Nevertheless, it seems highly unlikely that this spatial difference accounts for the inability of Yki to induce dedifferentiation in *rbf* mutant cells since photoreceptor recruitment and specification within the MF is not affected in *rbf wts* double mutant tissue. Therefore, we concluded that although overexpression of Yki recapitulates the tissue overgrowth phenotype of the Hippo pathway mutants, it is insufficient to mimic the dedifferentiation defects of *rbf wts* mutant photoreceptors.

## Discussion

The normal process of differentiation is accompanied by a declining plasticity of cells that commit to lineage-specific fates. In non-pathological conditions, this eventually culminates in an irreversible state of terminal differentiation. We found that in *rbf wts* double mutant cells this irreversibility is lost. It is widely acknowledged that inactivation of the pRB pathway is an obligatory early event in tumorigenesis, while the tumor suppressive function of pRB is usually attributed to its role in promoting cell cycle exit [7]. Surprisingly, we found that dedifferentiation of *rbf wts* double mutant cells is not due to a failure to exit the cell cycle. Thus, our results illuminate a novel function of pRB, guarding the differentiated state of a cell, and suggest that it is separable and distinct from the cell cycle exit control. Dedifferentiation is an important topic in cancer biology. It is well known that tumors containing cells with the morphological features of progenitor cells are generally more malignant than those resembling differentiated cells. Thus, tumor aggressiveness often correlates inversely with the extent of differentiation within a tumor. One implication of this work is that the loss of *Rb* may sensitize cells to dedifferentiation. More broadly, our results suggest that the concomitant loss of both the pRB and Hippo pathways allows cells to revert back to a progenitor-like state and therefore to have an increased potential to contribute to tumor growth.

In this work we employed the *Drosophila* retina to study the role of the pRB and Hippo pathways in differentiation. In the *Drosophila* retina, the developmental specification and recruitment of uncommitted cells is required for terminal neuronal differentiation. We found that both cell type specific (Sen and Ro) and more general (Elav and Nrg) neuronal markers are continuously lost over time in the *rbf wts* mutant tissue. Since improper recruitment impacts terminal differentiation then the loss of neuronal markers could be an indirect consequence of defects in recruitment. However, our analysis of R8 cell recruitment in *rbf wts* double mutant clones strongly argues against such an explanation. Specifically the expression of Atonal, a pro-neural gene that determines specification of the pre-R8 cell, and Scabrous, a secreted protein required for proper pattern formation of ommatidia to occur [35], were initiated and correctly resolved in *rbf wts* double mutant clones. Additionally, our data are distinct from previously reported R8 specific differentiation defects. For example, a reduction in the number of Sens positive cells towards the posterior has been previously described in *egfr* mutant clones. However, the *egfr* mutant phenotype can be rescued by overexpression of p35 suggesting that in this setting R8 cells are eliminated by apoptosis [45]. In contrast, there is no apoptosis in *rbf wts* double mutant tissue and the loss of R8 cells occurred even in the presence of p35. In another example, a pre-R8 cell forms in *sens* mutant ommatidia, but never a mature R8 cell. As a result, the pre-R8 cell switches fate to become an R2/R5 cell [46]. This is clearly not the case in *rbf wts* double mutant clones as mature R8 cells expressing the late neuronal marker Elav were present. This previous work further suggested that R-cell type specific signaling events are mutually exclusive from R-cell recruitment and resolution [46]. Our work expands upon this idea to suggest that following R-cell recruitment the pRB and Hippo pathways play a key role in maintaining the pro-neural program innate to each cell type in order to prevent the cell from dedifferentiating.

The fact that an *rbf* mutation leads to the loss of differentiation markers itself is not unexpected, since, for instance, *Rb^-/-^* mouse embryos display a variety of differentiation defects [47]–[49]. However, what constitutes a novel finding is that a progressive loss of differentiation markers as seen in *rbf wts* double mutant cells has not been previously reported in *Rb^-/-^* mouse knockouts. Similarly, tissue specific ablation of *Rb* in terminally differentiated cells *in vivo* is not accompanied by dedifferentiation. For example, during mouse inner ear development, *Rb^-/-^* hair cells undergo inappropriate cell divisions while remaining fully differentiated [11]. In *Drosophila*, cells that are double mutant for *rbf* and *dacapo*, a p21 homolog, differentiate into photoreceptors and, at the same time, undergo further cell divisions without the loss of the late neuronal marker Elav [18]. Even in tumorigenic settings *Rb^-/-^* cells do not undergo dedifferentiation, as has been demonstrated by analysis of *Rb^-/-^ p130^-/-^ p107^+/-^* horizontal interneurons. These cells inappropriately re-enter the cell cycle, clonally expand, and form metastatic retinoblastoma in mice, yet they remain highly differentiated cells [50]. These studies concluded that pRB operates *in vivo* at the point of terminal cell cycle exit. Our results suggest that, additionally, the pRB pathway, in cooperation with the Hippo pathway, has an important function in maintenance of a differentiated state.

One question that particularly interested us was whether the inappropriate proliferation of *rbf wts* double mutant photoreceptors triggers dedifferentiation. Earlier studies showed that inappropriate proliferation interferes with differentiation in cultured cells; and that the majority of differentiation defects in *Rb^-/-^* mouse knockouts appear to be an indirect consequence of defects in cell cycle exit and apoptosis or are a reflection of an extraembryonic function of *Rb* [14]–[16], [51], [52]. Therefore it was surprising that the *rbf wts* double mutant photoreceptors dedifferentiate in the complete absence of cell proliferation indicating that inappropriate cell cycle re-entry by itself is not sufficient to cause dedifferentiation. Consistently, driving *rbf* mutant photoreceptors into the cell cycle by overexpression of *yki* (this study) or by a concomitant loss of *dacapo* [18] does not cause dedifferentiation. Thus, the function of *rbf* in the maintenance of a differentiated state is unrelated and independent of the role of *rbf* during the cell cycle exit.

Why combined inactivation of the Hippo and pRB pathways causes dedifferentiation is not known. We disfavor an explanation that this is merely a cumulative effect of inactivation of two negative regulators of cell proliferation. Although we haven't extensively tested other tumor suppressor genes, at least the loss of *tsc1* failed to induce dedifferentiation of *rbf* mutant cells. Intriguingly, it has been previously shown that *hpo* and *wts* function in differentiated photoreceptors to regulate stable fate choice of the R8 cell subtypes [24]. Thus, dedifferentiation of *rbf wts* double mutant cells may reflect a specific functional overlap between pRB and Hippo pathways in neuronal cells.

The idea that the Hippo pathway has a postmitotic function is further supported by the fact that while *yki* expression readily drives differentiated *rbf* mutant cells into the cell cycle, the presence of a functional Warts kinase in these cells protects them from dedifferentiating. One implication of this result is that other effector(s) of the Hippo pathway may cause dedifferentiation of *rbf* mutant cells. Several studies have described Yki independent functions of Wts, such as the regulation of dendritic tiling and maintenance [25] and control of autophagic cell death in salivary glands [26]. It is worth noting that in contrast to *rbf wts* double mutants, photoreceptor differentiation occurs normally in *wts de2f1 de2f2* triple mutants [40]. This suggests that the differentiation phenotype observed in *rbf wts* double mutants is likely to reflect an E2F independent function of *rbf*. Although the analysis of differentiation in *rbf wts de2f1 de2f2* quadruple mutant cells will be needed to confirm this point, our data raise an intriguing possibility that the convergence of the pRB and Hippo pathways in maintaining a differentiated state lies outside of their conventional roles to restrain the activities of E2F and Yki respectively.

The process of cell-specific lineage commitment and terminal differentiation is accompanied by stabilization and maintenance of each cell-type specific transcription program. This is achieved by a progressive restriction of chromatin accessibility to genes that promote proliferation and, conversely, increasing chromatin accessibility to tissue-specific genes. It is well established that the Polycomb group (PcG) genes play key roles in “locking” the chromatin state during cell specification and differentiation [53], [54]. Intriguingly, both the pRB and Hippo pathways were linked to the regulation of chromatin structure in *Drosophila*. For example, RBF was shown to directly regulate chromatin condensation [55], while the Hpo and Wts kinases genetically and physically interact with Polycomb Group proteins (PcG) [25]. Thus, it is tempting to speculate that the dedifferentiation of photoreceptors in *rbf wts* double mutants could be the result of alterations in gene expression due to aberrant epigenetic changes in cells lacking functional pRB and Hippo pathways.

## Materials and Methods

### Fly stocks

All crosses were done at room temperature unless otherwise stated.

*rbf1^120a^ ey-FLP / Y; 82BFRT de2f1^729^ wts^X1^ / 82B FRT [Ubi-GFP]*

*rbf1^120a^ ey-FLP / Y; 82BFRT wts^X1^ / 82B FRT [Ubi-GFP]*

*rbf1^120a^ ey-FLP / Y; 42DFRT hpo^MGH4^ / 42DFRT [Ubi-GFP]*

*rbf1^120a^ ey-FLP / Y; GMR-Gal4,UAS-p35 / UAS-Yki^S168A^*

*rbf1^120a^ ey-FLP / +; GMR-Gal4,UAS-p35 / UAS-Yki^S168A^*

*rbf1^120a^ ey-FLP / Y; GMR-Gal4,UAS-p35 / +; 82BFRT wts^X1^ / 82B FRT [Ubi-GFP]*

*rbf1^14^ FRT19A / [Ubi-GFP] FRT 19A; ey-FLP / +; 82BFRT wts^X1^ / 82B FRT [Ubi-GFP]*

*rbf1^14^ FRT19A / [Ubi-GFP] FRT 19A; ey-FLP / +*

*ey-FLP / +; 82BFRT wts^X1^ / 82B FRT [Ubi-GFP]*

*rbf1^120a^ hs-FLP / Y; 82BFRT wts^X1^ / 82B FRT [Ubi-GFP]*

*rbf1^120a^ hs-FLP / Y; 82BFRT tsc^f01910^ / 82B FRT [Ubi-GFP]*

*rbf1^120a^ hs-FLP / Y; 82BFRT tsc^3^ / 82B FRT [Ubi-GFP]*

*hs-FLP / Y; 82BFRT tsc^f01910^ / 82B FRT [Ubi-GFP]*

*hs-FLP / Y; 82BFRT tsc^3^ / 82B FRT [Ubi-GFP]*

*hs-FLP / Y; 82BFRT wts^X1^ / 82B FRT [Ubi-GFP]*

### Heatshock treatment

To generate adult and larval *rbf wts* and *rbf tsc* double mutant tissue, and *wts* or *tsc* single mutant tissue, clones were induced 48 hr AED for 20 minutes at 37°C, and then larvae were grown at 25°C.

### Immunohistochemistry

Antibodies used were as follows: guinea pig anti-Senseless 1∶2000 (from H. Bellen), rabbit anti-Yki 1∶800 (from K. Irvine), rat anti-ELAV 1∶200 (DSHB), mouse anti-Nrg 1∶100 (DSHB), mouse anti-Eya 1∶100 (DSHB), mouse anti-Scabrous 1∶30 (DSHB), mouse anti-Rough 1∶100 (DSHB), mouse anti-Cut 1∶200 (DSHB), rabbit anti-Atonal 1∶2000 (from Y. Jan), rabbit anti-C3 (Cleaved Caspase3) 1∶100 (Cell Signaling), mouse anti-BrdU 1∶50 (Beckton Dickinson), rabbit anti-phosH3 1∶175 (Upstate), mouse anti-RBF1 1∶20, and Cy3, Cy5 conjugated anti-mouse, anti-rabbit, anti-rat, and anti-guinea pig secondary antibodies (Jackson Immunolaboratories).

Larval and pupal tissues were fixed in 4% formaldehyde +1X phosphate-buffered saline for 35 minutes on ice, washed in 1X phosphate-buffered saline two times for 5 min on ice, then permeabilized in 1X phosphate-buffered saline +0.3% Triton-X100 three times for 5 minutes each, and then incubated with antibodies overnight at 4°C in phosphate-buffered saline, 10% normal goat serum, and 0.3% Triton-X100. After the overnight incubation, samples were washed in 1X phosphate-buffered saline + 0.1% Triton-X100 three times for 5 minutes each at room temperature. Samples were then incubated with appropriate conjugated secondary antibodies for 1 hour at room temperature in phosphate-buffered saline, 10% normal goat serum, and 0.3% Triton-X100. Finally, samples were washed five times for 5 minutes each at room temperature in 1X phosphate-buffered saline + 0.1% Triton-X100 before being stored in glycerol + antifade reagents and then mounted on glass slides.

To detect S phases dissected larval eye discs were labeled with BrdU for 2 hrs at room temperature and then the eye discs were fixed overnight in 1.5% formaldehyde + 1X phosphate-buffered saline + 0.2% Tween−20 at 4°C. Samples were then digested with DNAase (Promega) treatment for 30 minutes at 37°C. Samples were then treated with primary and secondary antibodies as described above. All immunoflourescence was done on a Zeis Confocal microscope and images were prepared using Adobe Photoshop CS4. All images are confocal single plane images unless otherwise stated as projection images.

### Quantification methods

#### Figure 1Ei

Percentages represent absolute values for each category (0, 1, 2 Senseless) per genotype (100% being the maximum combined for all three categories). Counting was done in 8 individual discs and total numbers of mutant clusters counted was 231 and 280 clusters for the wild-type. A cluster was counted if it was within the area of the eye disc from 3 Elav positive columns behind the morphogenetic furrow to the posterior edge of the disc. In this position of a wild-type disc refinement, resolution, and recruitment of the R8 cell has ceased. A Student's *t*-Test using the parameters of two samples, one-tail, and unequal variance was used to determine statistical significance.

#### Figure 3D

Percentages represent absolute value for each category per genotype (100% being the total of the combined values for all eight categories). Counting was done in 8 individual discs for the *rbf wts* double mutant and 6 individual discs for each other genotype. Total numbers of clusters counted was as follows:

*rbf wts* double mutant was 231

*rbf* single mutant 140

*wts* single mutant 90

wild-type 138

A cluster was counted if it was within the area of the eye disc from 3 Elav positive columns behind the morphogenetic furrow to the posterior edge of the disc. In this position of a wild-type disc the recruitment of the first 5 R-cells has been achieved and the ensuing R6, R1, and R7 cell will take place. So a range of cluster size of will be between five and seven Elav positive cells. A Student's *t*-Test using the parameters of two samples, one-tail, and unequal variance was used to determine statistical significance between each mutant genotype and wild-type.

## Supporting Information

Figure S1 *rbf hpo* double mutants have defects in differentiation. All images are projection images. (A) Photoreceptors differentiate normally in clones of *hpo^MHG4^* mutant cells, as seen by Senseless (Sens) (red) and Elav (blue) expression. (B) The number of Sens positive cells is reduced in the posterior of the *rbf^120a^ hpo^MHG4^* mutant clones. White arrows point to Elav positive clusters of cells that lack any Sens positive cell. Elav expression reveals an incomplete complement of photoreceptors in the posterior of the double mutant tissue.
(1.22 MB TIF)

Figure S2 *rbf wts* double mutants have defects in differentiation. All images are projection images. Reduced number of Elav (A) and Sens (B) positive cells in the posterior of the *rbf^14^ wts^x1^* double mutant tissue. RBF antibody was used to detect *rbf* mutant cells. (A) Clones of four different genotypes generated by the FLP/FRT system can be found: *rbf^+/+^ wts^+/+^* is marked by the presence of RBF (red) and GFP (green) and is outlined in white; *rbf^+/+^ wts^-/-^* is marked by the presence of RBF and reduced level of GFP and is outlined in yellow; *rbf^-/-^ wts^+/+^* is marked by the presence of GFP and the absence of RBF and is outlined in magenta; *rbf^-/-^ wts^-/-^* is marked by both the absence of GFP and the absence of RBF. (B) The eye disc is almost entirely comprised of *rbf^14^ wts^x1^* double mutant tissue. The wild-type tissue can be identified in the upper portion of the image by the presence of GFP and a normal spacing between Sens (red) positive cells.
(1.52 MB TIF)

Figure S3 *rbf wts* double mutant ommatidial cells can be refined and recruited properly, but fail to maintain a differentiated state. All images are projection images. R2/R5 photoreceptors differentiate following differentiation of R8. Expression of the R8 marker Sens (blue) and the R2/R5 marker Ro (red) in a wild-type eye disc (A) and in eye discs containing clones of *rbf^120a^ wts^x1^* (B) and *rbf^14^ wts^x1^* (C) double mutant cells. In a wild-type disc, a pair of Ro positive cells can be found next to a single Sens positive cell. The number of Ro positive cells is reduced in the posterior of the double mutant clone. Multiple examples of a single Ro positive cell in *rbf^14^ wts^x1^* double mutant tissue can be identified and are pointed at by arrows in (C).
(1.99 MB TIF)

Figure S4 Lack of apoptosis in the posterior of *rbf wts de2f1* triple mutant tissue. (A) No apoptotic cells were detected in the posterior of *rbf^120a^ wts^x1^ de2f1^729^* triple mutant cells. (B) A known Hippo pathway target dIAP1 remains elevated in *rbf^120a^ wts^x1^ de2f1^729^* triple mutant cells.
(1.25 MB TIF)
