# Supplementary figures and images for: Combined Inactivation of pRB and Hippo Pathways Induces Dedifferentiation in the Drosophila Retina
Source: PLoS Genet. 2010 Apr 22;6(4):e1000918. doi: 10.1371/journal.pgen.1000918 (PMC2858677; doi:10.1371/journal.pgen.1000918)

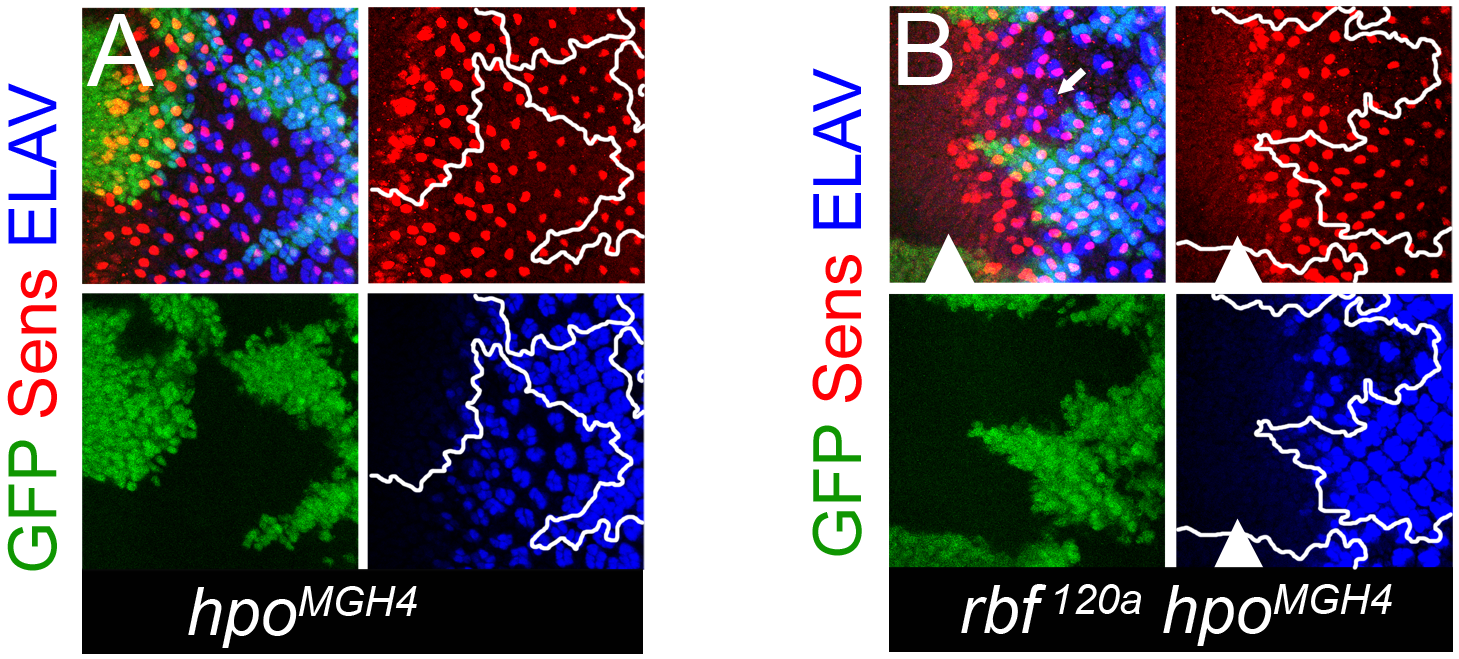

Supplement: Figure S1 — rbf hpo double mutants have defects in differentiation. All images are projection images. (A) Photoreceptors differentiate normally in clones of hpoMHG4 mutant cells, as seen by Senseless (Sens) (red) and Elav (blue) expression. (B) The number of Sens positive cells is reduced in the posterior of the rbf120a hpoMHG4 mutant clones. White arrows point to Elav positive clusters of cells that lack any Sens positive cell. Elav expression reveals an incomplete complement of photoreceptors in the posterior of the double mutant tissue. (1.22 MB TIF) [file pgen.1000918.s001.tif]

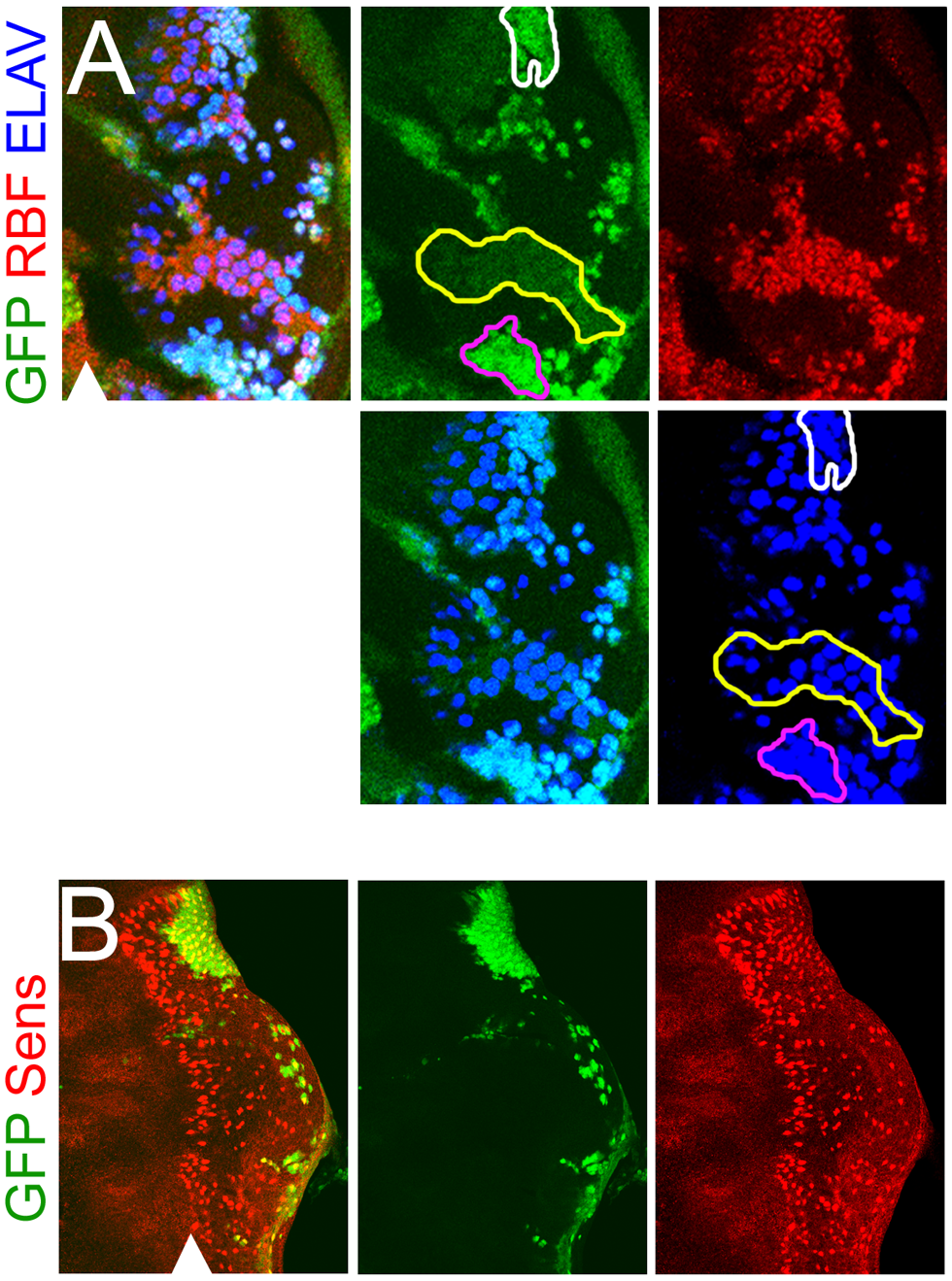

Supplement: Figure S2 — rbf wts double mutants have defects in differentiation. All images are projection images. Reduced number of Elav (A) and Sens (B) positive cells in the posterior of the rbf14 wtsx1 double mutant tissue. RBF antibody was used to detect rbf mutant cells. (A) Clones of four different genotypes generated by the FLP/FRT system can be found: rbf+/+ wts+/+ is marked by the presence of RBF (red) and GFP (green) and is outlined in white; rbf+/+ wts-/- is marked by the presence of RBF and reduced level of GFP and is outlined in yellow; rbf-/- wts+/+ is marked by the presence of GFP and the absence of RBF and is outlined in magenta; rbf-/- wts-/- is marked by both the absence of GFP and the absence of RBF. (B) The eye disc is almost entirely comprised of rbf14 wtsx1 double mutant tissue. The wild-type tissue can be identified in the upper portion of the image by the presence of GFP and a normal spacing between Sens (red) positive cells. (1.52 MB TIF) [file pgen.1000918.s002.tif]

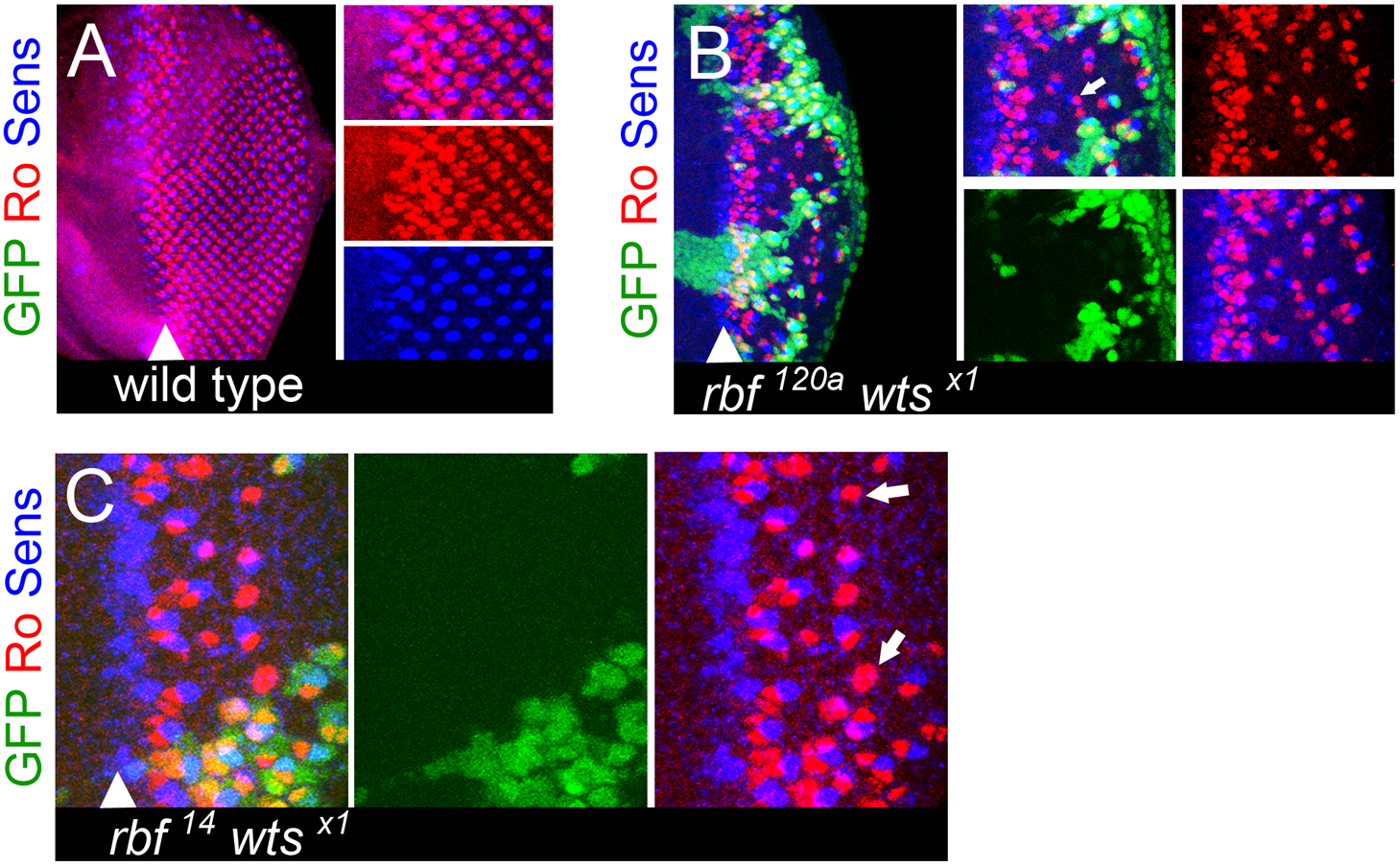

Supplement: Figure S3 — rbf wts double mutant ommatidial cells can be refined and recruited properly, but fail to maintain a differentiated state. All images are projection images. R2/R5 photoreceptors differentiate following differentiation of R8. Expression of the R8 marker Sens (blue) and the R2/R5 marker Ro (red) in a wild-type eye disc (A) and in eye discs containing clones of rbf120a wtsx1 (B) and rbf14 wtsx1 (C) double mutant cells. In a wild-type disc, a pair of Ro positive cells can be found next to a single Sens positive cell. The number of Ro positive cells is reduced in the posterior of the double mutant clone. Multiple examples of a single Ro positive cell in rbf14 wtsx1 double mutant tissue can be identified and are pointed at by arrows in (C). (1.99 MB TIF) [file pgen.1000918.s003.tif]

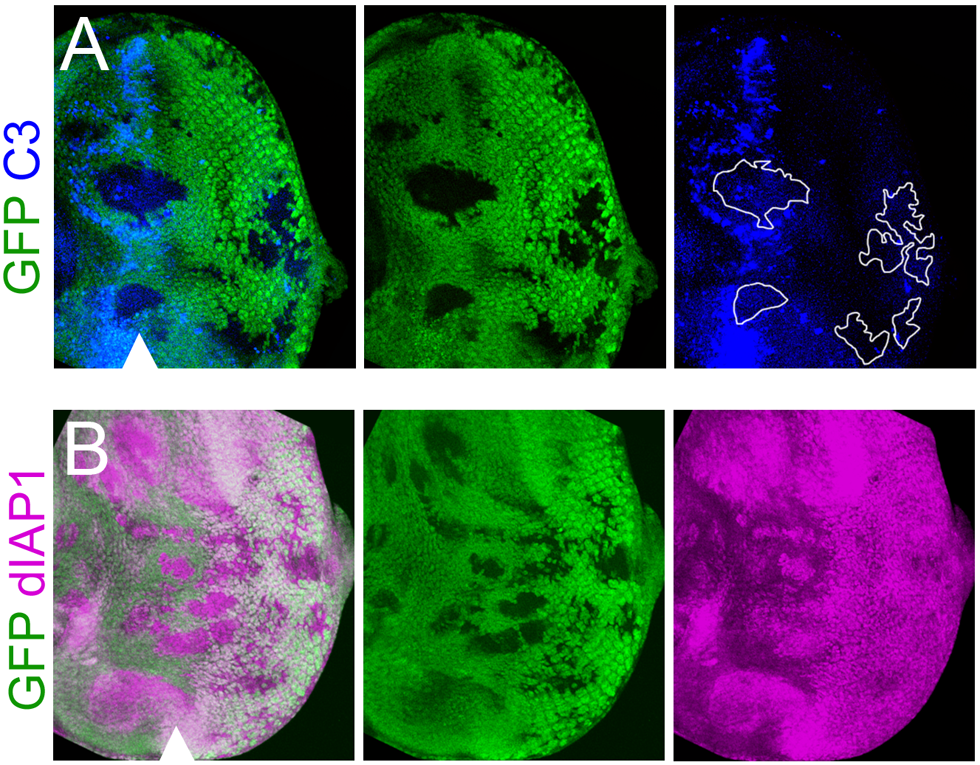

Supplement: Figure S4 — Lack of apoptosis in the posterior of rbf wts de2f1 triple mutant tissue. (A) No apoptotic cells were detected in the posterior of rbf120a wtsx1 de2f1729 triple mutant cells. (B) A known Hippo pathway target dIAP1 remains elevated in rbf120a wtsx1 de2f1729 triple mutant cells. (1.25 MB TIF) [file pgen.1000918.s004.tif]
